# Extract of *Monascus purpureus* CWT715 Fermented from Sorghum Liquor Biowaste Inhibits Migration and Invasion of SK-Hep-1 Human Hepatocarcinoma Cells

**DOI:** 10.3390/molecules21121691

**Published:** 2016-12-08

**Authors:** Wen-Teish Chang, Cheng-Hung Chuang, Wan-Ju Lee, Chin-Shiu Huang

**Affiliations:** 1 Department of Food Science, National Penghu University of Science and Technology, Penghu Hsien 88046, Taiwan; changcc@gms.npu.edu.tw; 2 Department of Nutrition, Hungkuang University, 1018 Sec. 6 Taiwan Boulevard, Taichung 43302, Taiwan; chchuang@hk.edu.tw; 3 Department of Health and Nutrition Biotechnology, Asia University, Taichung 41354, Taiwan; amy04221008@gmail.com; 4 Department of Medical Research, China Medical University Hospital, China Medical University, Taichung 40402, Taiwan

**Keywords:** Kinmen sorghum liquor waste, *Monascus purpureus*, anti-metastasis, nm23-H1, hepatocarcinoma

## Abstract

Liver cancer is the most endemic cancer in a large region of the world. This study investigated the anti-metastatic effects of an extract of *Monascus purpureus* CWT715 (MP) fermented from sorghum liquor biowaste and its mechanisms of action in highly metastatic human hepatocarcinoma SK-Hep-1 cells. Kinmen sorghum liquor waste was used as the primary nutrient source to produce metabolites (including pigments) of MP. In the presence of 10 µg/mL MP-fermented broth (MFB), the anti-invasive activity increased with increasing fermentation time reaching a maximum at six days of fermentation. Interestingly, MFB also produced maximal pigment content at six days. Treatment for 24 h with MFB (10–100 µg/mL) obtained from fermentation for six days significantly inhibited cell migration and invasion, and these effects were concentration-dependent. MFB also significantly enhanced nm23-H1 protein expression in a concentration-dependent manner, which was highly correlated with migration and invasion. These results suggest that MFB has significant anti-migration and anti-invasion activities and that these effects are associated with the induction of nm23-H1 protein expression.

## 1. Introduction

Hepatocellular carcinoma is the second leading cause of cancer death in Taiwan and is endemic in a large portion of the world. Tumor metastasis, cancer cells from their primary site to a distant organ, is a major cause of death for cancer patients. Cancer cell metastasis is characteristic of tumor progression involving complex processes including the detachment of tumor cells, ability to dissolve the basement membrane and the extracellular matrix, and to migrate through the extracellular matrix (ECM) [1], for which many steps involving accumulation of genetic changes and destruction of biological barriers are required. Altered expression of the metastasis-related gene is considered to play a pivotal role. The *nm23* gene, a metastasis suppressor gene, has been demonstrated to inhibit cell motility and invasion [2,3,4,5]. Eight human *nm23* genes have been characterized so far, of which the *H1* gene is the most closely correlated with the metastatic phenotype in several types of cancer such as hepatocarcinoma [6,7]. Furthermore, one critical characteristic that metastatic cancer cells have acquired is the degradation of the basement membranes and ECM. Matrix metalloproteinases (MMPs) efficiently degrade all known components of the ECM, which results in tumor metastasis [8]. MMP-2 (72-kDa) and MMP-9 (92-kDa) have been implicated in malignant tumor progression, partly because they degrade collagen types IV, V, VII, and X; fibronectin; and gelatin of basement membranes [9,10]. To find novel natural compounds for blocking these biological events is an important topic in cancer research.

*Monascus* species produce valuable metabolites, including pigments, γ-aminobutyric acid, and monacolin K, which are used as colorants, medcines, and health supplements that are known to improve health and to prevent and remedy various diseases such as cancer [11,12,13,14]. Pigments from *Monascus* metabolites, including ankaflavin [15], monascin [16], and rubropunctatin [17], have been suggested to account for the observed anti-tumor effects. Hong et al. [18] have shown that the Chinese red yeast rice, a *Monascus* species, significantly reduces tumor volumes of androgen-dependent and androgen-independent prostate xenograft tumors in SCID mice. Zheng et al. [19] have suggested that the *Monascus* pigments exert cytotoxicity in human cancer cells (SH-SY5Y, HepG2, HT-29, BGC-823, AGS, and MKN45). Moreover, Ho and Pan [12] have found that the *Monascus* metabolite monacolin K reduces tumor progression and metastasis in Lewis lung carcinoma (LLC) cells. However, the mechanism underlying the anti-metastatic action by the *Monascus* metabolite is unclear. In the present study, we employed a highly invasive hepatocarcinoma, the SK-Hep-1 cell line, to examine the effects of the fermentative extract of *M. purpureus* CWT715 (MP) on cell migration and invasion and the possible underlying mechanisms.

## 2. Results and Discussion

### 2.1. Effect of Fermentation Time on Pigment Production MP-Fermented Broth (MFB)

As shown in Table 1, pigment production of MFB increased with fermentation time. Maximum pigment production was obtained after six days of fermentation, after which pigment production decreased slightly. Therefore, MFB exhibited time-dependent pigment production, and the pigments were stable during the seven-day fermentation period. In our previous study, the total phenol was also increased after fermentation in a similar manner, and the maximum total phenol (15.3 ± 0.5 mg/g) was reached at six days of fermentation [20].

### 2.2. Effects of Fermentation Time of MFB on In Vitro Cell Invasion

**Figure 1:**
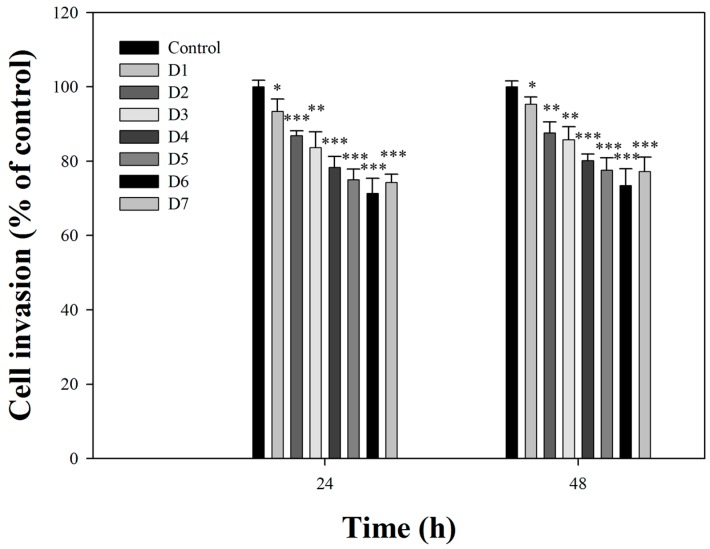
Anti-invasive activity throughout time of incubation of final effluent treated with MFB in SK-Hep-1 cells. Cells (1 × 10^4^) were incubated for 24 and 48 h with different fermentation time MFB (0–7 days, 10 µg/mL). D1, D2, D3, D4, D5, D6, and D7 represent 1, 2, 3, 4, 5, 6, and 7 days. Values are means ± SD, *n* = 3; significant difference from control value was indicated * *p* ≤ 0.05, ** *p* ≤ 0.01, and *** *p* ≤ 0.001. Value at the same dose had no significant difference between 24 and 48 h incubation.

The effect of MFB obtained on one through seven days on the invasion of SK-Hep-1 cells was investigated (Figure 1). Incubation of SK-Hep-1 cells with MFB (10 µg/mL) for 24 h and 48 h revealed that the anti-invasive activity of MFB increased with increasing fermentation time, reaching a maximum on day six after fermentation. Based on these findings, MFB obtained from six-day fermentation was chosen for the following studies. In addition, there was no significant difference between 24 and 48 h incubation. Interestingly, contents of pigments were highly associated with anti-invasive activity (at 24 h treatment: yellow pigment, *r*^2^ = 0.91; orange pigment, *r*^2^ = 0.91; red pigment, *r*^2^ = 0.91). These results indicate that anti-invasive activity of MFB is associated with pigment content.

Many studies have indicated that the pigments in the metabolites of *Monascus* species produce anticancer effects [15,16,17,18]. Among these pigments, rubropunctatin is most efficient in the induction of apoptosis in several human cancer cells [18]. Monascuspiloin, a yellow pigment separated from metabolites of *Monascus*, has also been reported to induce apoptosis in human prostate cancer cells through the PI3K/Akt and MAPK signaling pathways [21]. The results of the current study demonstrated that the pigment content of the culture medium obtained from fermentation of KSL by CWT715 increased with the duration of culture, and the anti-invasive activity also increased. Our results are in accord with those of Ho et al. [22], which reported that *Monascus*-fermented rice extract downregulated VEGF-mediated SW 620 cell invasion. Moreover, ethanol extracts of *Monascus*-fermented rice has been found to decrease the number of metastatic colonies in LLC-bearing mice [12].

### 2.3. Effects of MFB on the Proliferation of SK-Hep-1 Cells

**Figure 2:**
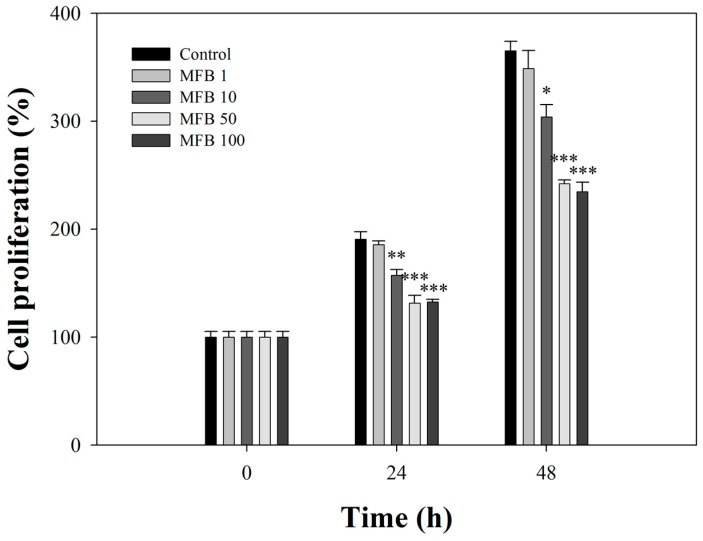
Effects of MFB on cell proliferation of SK-Hep-1 cells. Cells (1 × 10^4^) were incubated with MFB (1–100 μg/mL) for 24 and 48 h. MFB1, MFB10, MFB50, and MFB100 represent 1, 10, 50, and 100 µg/mL, respectively. Values (means ± SD, *n* = 3) at the same time point significant difference from control value was indicated * *p* ≤ 0.05, ** *p* ≤ 0.01, and *** *p* ≤ 0.001.

The broth (100 µg/mL) significantly inhibited the proliferation of SK-Hep-1 cells in a concentration-dependent manner by 31% (*p* < 0.001) and 36% (*p* < 0.001) at 24 h and 48 h of incubation, respectively (Figure 2). This activity was not attributed to necrosis because the amount of lactate dehydrogenase released from cells incubated with the broth was not significantly different from that of controls (Supplementary Materials Figure S1). These results suggest that MFB may be responsible for the anti-proliferative activity observed. Several studies have reported that *Monascus* metabolites have been shown to exert anti-proliferative and anti-tumor capacities in various cancers [15,16,17,18].

### 2.4. Effect of MFB on In Vitro Invasion and Migration of SK-Hep-1 Cells

**Figure 3:**
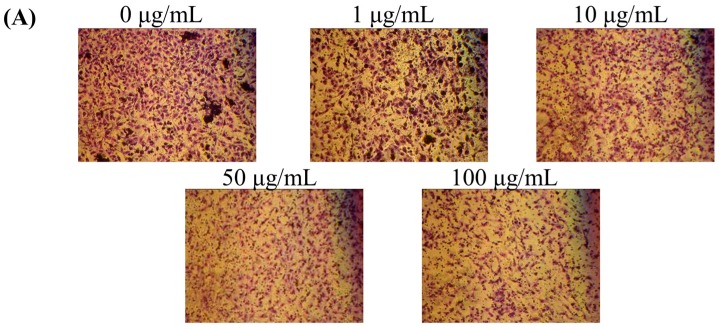

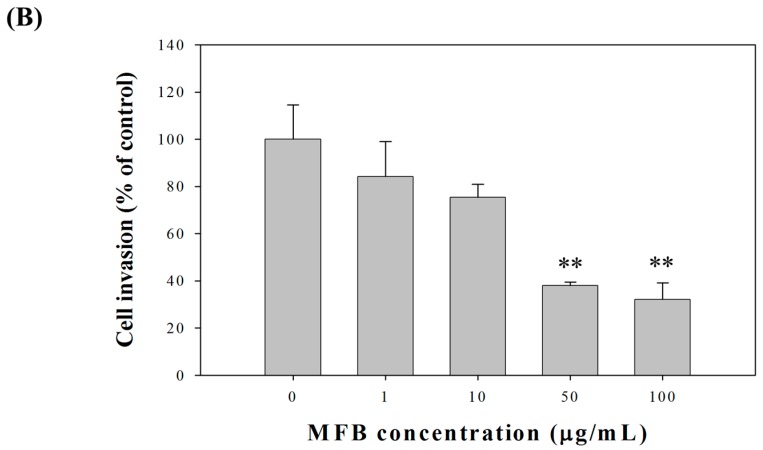
Effects of MFB on cell invasion of SK-Hep-1 cells. Cells (5 × 10^4^) were incubated with MFB (1–100 μg/mL) for 24 h. Representative phase contrast photomicrographs (100× magnification) were taken at 24 h (**A**) and invasion ability was quantified (**B**) by counting the number of cells that invaded to the underside of the membrane. Values are means ± SD, *n* = 3; significant difference from control value was indicated ** *p* ≤ 0.01.

**Figure 4:**
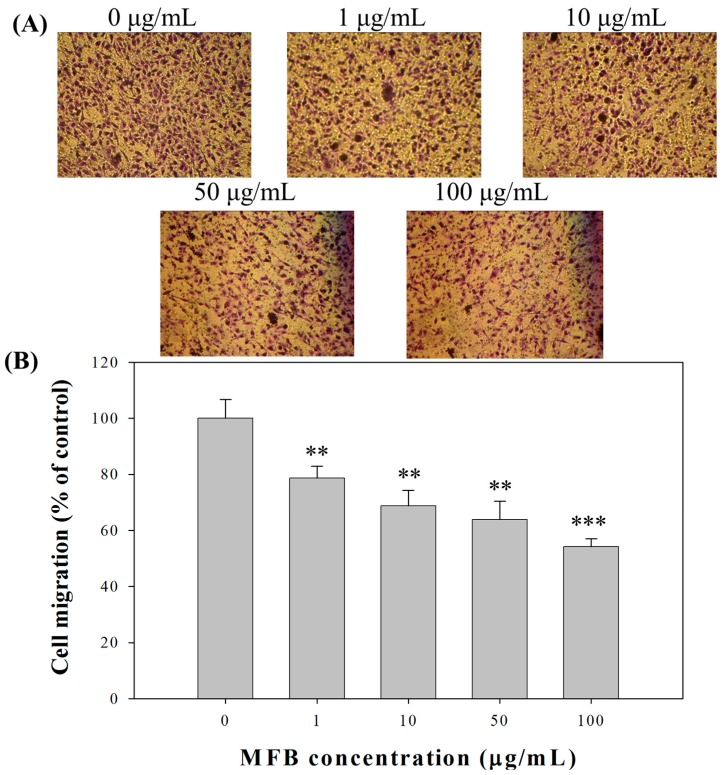
Effects of MFB on cell migration of SK-Hep-1 cells. Cells (5 × 10^4^) were incubated with MFB (1–100 μg/mL) for 24 h. Representative phase contrast photomicrographs (100× magnification) were taken at 5 h (**A**) and migration ability was quantified (**B**) by counting the number of cells that invaded to the underside of the membrane. Values are means ± SD, *n* = 3; significant difference from control value was indicated ** *p* ≤ 0.01, and *** *p* ≤ 0.001.

Because MFB inhibited cell proliferation, the number of cells had to be adjusted to an equal number (5 × 10^5^ cells/mL) before the determination of cell migration and cell invasion to avoid interference by cell numbers. Under this condition, we found that MFB (10–100 µg/mL) significantly inhibited the cell invasion of SK-Hep-1 cells in a concentration-dependent manner at 24 h of incubation, with 68% (*p* < 0.001) inhibition of cell invasion at a concentration of 100 µg/mL MFB (Figure 3). The effects of MFB on cell migration were similar to those on cell invasion—i.e., MFB significantly inhibited cell migration—and the effects were concentration-dependent, with 46% (*p* < 0.001) inhibition of cell migration at a concentration of 100 µg/mL MFB (Figure 4). The *Monascus* metabolites have been shown to inhibit metastasis in cancer cells, such as monacolin K [12] and dimerumic acid [13]. For instance, monacolin K has been shown to inhibit metastasis in Lewis lung carcinoma-bearing mice.

### 2.5. Upregulation of nm23-H1 by MFB at the Protein Level 

**Figure 5:**
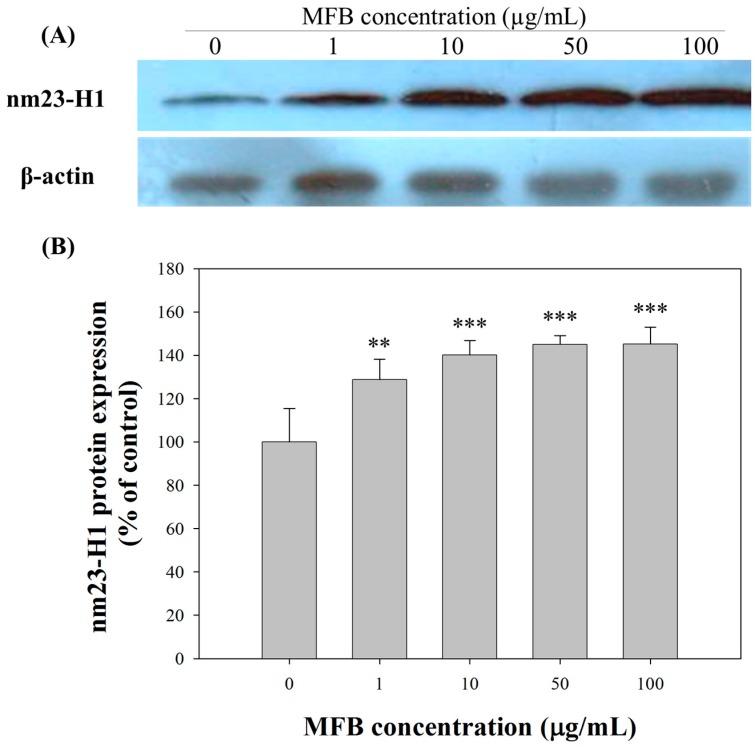
Effects of MFB on nm23-H1 protein expression in SK-Hep-1 cells. Cells were incubated with MFB at various concentrations (1–100 μg/mL) for 24 h. (**A**) Western blots of nm23-H1and β-actin; (**B**) Densitometric analysis of (**A**). For the loading control, expression levels of β-actin were analyzed by using the same lysate. Values are means ± SD, *n* = 3; significant difference from control value was indicated ** *p* ≤ 0.01, and *** *p* ≤ 0.001.

The expression of nm23-H1 protein was affected by MFB in a concentration-dependent manner (Figure 5). At 100 µg/mL, MFB induced the highest expression of nm23-H1 protein (240% ± 8%, *p* < 0.001). In addition, nm23-H1 protein expression correlated negatively with migration (*r^2^* = 0.95, *p* = 0.002) and invasion (*r*^2^ = 0.85, *p* = 0.038) in SK-Hep-1 cells. Thus, the decrease in the metastasis-associated phenotypes, such as cell migration and cell invasion, induced by MFB treatment may at least be partially mediated by its upregulation of nm23-H1 expression. Several compounds have been reported to elevate Nm23H1 expression in vitro, which may lead to inhibition of cell migration and invasion [23].

Nm23-H1 has been reported to negatively regulate cell migration and tumor metastasis by modulating the activity of Rho GTPase family members, such as Cdc42, Rac 1, and Rho [24,25]. Both Cdc42 and Rac1 promote actin polymerization at the leading edge, and thereby the formation of filopodia and larnellipodia are required for carcinoma migration and invasion [26]. Rho induces the assembly and contraction of the actomyosin fibers, which contributes to pulling the trailing edge forward during migration [27].

### 2.6. Effects of MFB on the Activities of MMP-9 and MMP-2

**Figure 6:**
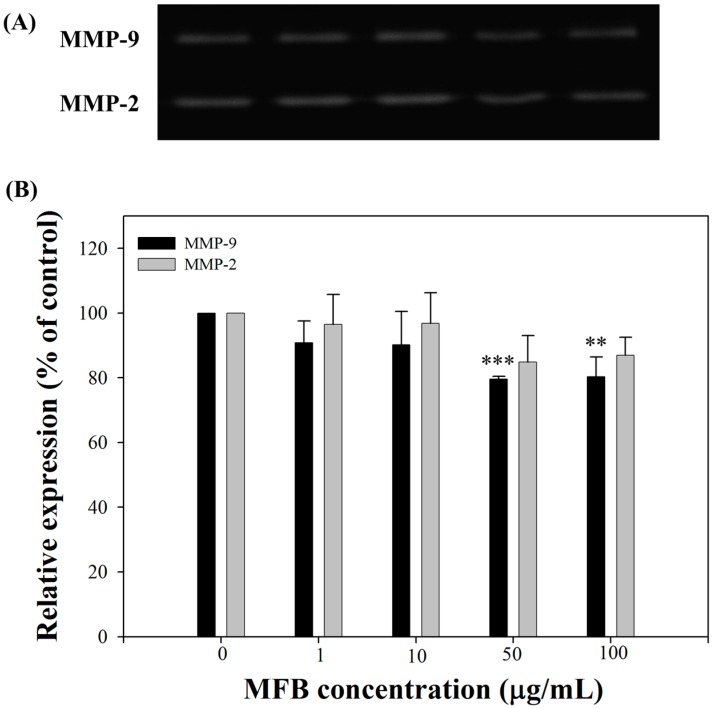
Effects of MFB on MMP-2 and MMP-9 activity in SK-Hep-1 cells. Cells (5 × 10^4^) were incubated with MFB for 24 h. (**A**) Zymography of MMP-2 and MMP-9; (**B**) Densitometric analysis of (**A**). Values are means ± SD, *n* = 3; significant difference from control value was indicated ** *p* ≤ 0.01, and *** *p* ≤ 0.001.

The zymography (Figure 6A) showed that incubation of MFB (1–100 µg/mL) for 24 h inhibited the activities of MMP-9 and MMP-2. At 50 µg/mL, MFB significantly suppressed the activities of MMP-9 by 20%, and slightly decreased the activities of MMP-2 by 15% (Figure 6B). Therefore, another possible anti-metastatic mechanism of MFB is through the inhibition of MMP-2 and MMP-9 activity, as MMPs have been shown to play an important role in the invasion and metastasis of cancerous cells [28,29]. The *Monascus* metabolite dimerumic acid has been reported to inhibit H_2_O_2_-mediated MMP-7 expression through attenuation of JNK and ERK signaling [13]. In addition, the expression of MMPs, including MMP-9 and MMP-2, has been shown to be down-regulated by nm23-H1 protein [30,31,32]. These results therefore indicate that the decreased expression of MMP-9 and MMP-2 by MFB was highly correlated with nm23-H1 expression (*r*^2^ = 0.88, *p* = 0.002 and *r*^2^ = 0.72, *p* = 0.005, respectively) in SK-Hep-1 cells.

## 3. Materials and Methods

### 3.1. Cell Line and Chemicals

The cell line SK-Hep-1 (BCRC 67005) was purchased from the Food Industry Research and Development Institute, Hsin Chu, Taiwan. All chemicals used were of reagent grade or higher. Distiller’s sorghum residue (DSR) was obtained as a byproduct from Kinmen Kaoliang Liquor, Kinmen County, Taiwan. Dulbecco’s minimal essential medium (DMEM), fetal bovine serum (FBS), trypsin, penicillin, streptomycin, sodium pyruvate, nonessential amino acid, and Giemsa stain were purchased from GIBCO/BRL (Rockville, MD, USA). Transwell chambers were obtained from Costar (Cambridge, MA, USA). Anti-nm23 mouse monoclonal antibody and anti-mouse IgG-HRP antibody were purchased from BD Co. (San Diego, CA, USA) and GeneTex, Inc. (Irvine, CA, USA), respectively.

### 3.2. Isolation and Screening of an Antioxidant-Producing Strain

Microorganisms isolated from soils obtained at different locations in Taiwan were further screened on agar plates containing 1% DSR, 0.1% K_2_HPO_4_, 0.05% MgSO_4_·7H_2_O, and 2% agar (pH 7.0) after two days of incubation at 30 °С. Colonies that grew well were isolated and retained for subsequent screening. The organisms obtained from the first screening were subcultured in 100 mL of DSR medium (1% DSR, 0.1% K_2_HPO_4_, and 0.05% MgSO_4_·7H_2_O) and incubated in shaking flasks at 180 rpm at 30 °С for seven days. Following centrifugation (8000× *g*, 4 °С, for 20 min, Hitachi CF15RXII, Tokyo, Japan), the antioxidative activities of the supernatants were measured. The strain that had the highest antioxidative activity, CWT715, was isolated [20], maintained on potato dextrose agar, and used in the study. The CWT715 strain was characterized by morphological observations and physiological features. It was identified by the Food Industry Research and Development Institute, Hsinchu, Taiwan.

### 3.3. Extract Preparation

*M. purpureus* CWT715 was inoculated in DSR medium and incubated at 30 °С for 1–7 days as described above. Furthermore, the mycelia and broth were collected and then freeze-dried. The dried powder was dissolved in phosphate-buffered saline, sonicated for 2 h, and centrifuged at 8000× *g* for 20 min. After centrifugation, the supernatant was collected and is referred to here as MFB. The concentration of extracellular pigments was estimated by measuring the absorbance of filtrates at 400 nm (yellow pigment), 470 nm (orange pigment), and 500 nm (red pigment), taking the dilution factor into consideration.

### 3.4. Cell Culture and Cell Proliferation

SK-Hep-1 cells were grown in DMEM containing 10% (*v*/*v*) FBS, 0.37% (*w*/*v*) NaHCO_3_, penicillin (100 unit/mL), and streptomycin (100 unit/mL) in a humidified incubator under 5% CO_2_ and 95% air at 37 °C. The cells were harvested at approximately 90% confluence (10^6^ cells/dish). Cell proliferation was mainly measured by 3-(4,5-dimethylthiazol-2-yl)-2,5-diphenol tetrazolium bromide colorimetric assay, and the trends in cell proliferation were confirmed by Trypan-blue assay. A stock MFB solution was freshly prepared before each experiment and was added to the culture medium at a final concentration of 1 µg/mL, 10 µg/mL, 50 µg/mL, or 100 µg/mL.

### 3.5. Cell Migration Assay

Tumor cell migration was assayed in transwell chambers (Costar) according to the methods of Repesh [33] with some modifications. Briefly, transwell chambers (Costar) with 6.5 mm polycarbonate filters of 8-µm pore size were used. After 24 h of incubation with MFB, Sk-Hep-1 cells (5 × 10^5^ cells/mL) were suspended in DMEM (100 µL, serum free), placed in the upper transwell chambers, and then incubated for 5 h at 37 °C. After 5 h of incubation at 37 °C, the cells on the upper surface of the filter were completely wiped away with a cotton swab. The cells on the lower surface of the filter were fixed in methanol, stained with Giemsa, and then counted under a microscope. For each replicate, the tumor cells in 10 randomly selected fields were determined, and the counts were averaged.

### 3.6. Cell Invasion Assay

The procedure reported by Repesh [33] for the cell invasion assay was similar to that for cell migration. The invasion of tumor cells was assessed in transwell chambers with a 6.5 mm diameter polyvinyl/pyrrolidone-free polycarbonate filter of 8-µm pore size. Each filter was coated with 100 µL of 1:20 diluted Matrigel in cold DMEM to form a thin continuous film on the top of the filter. The number of cells was adjusted to 5 × 10^5^ cells/mL, and a 100-µL aliquot containing 5 × 10^4^ cells was added to each of the triplicate wells in DMEM containing 10% FBS. After 24 h of incubation, the cells were stained and counted as described above, and the number of cells invading the lower side of the filter was measured as the invasive activity.

### 3.7. Gelatin Zymography

MMP-2 and MMP-9 activity were assayed by using gelatin zymography according to the methods described previously [7]. The cells (5 × 10^4^ cells/mL) were treated with MFB for 24 h in DMEM containing 10% (*v*/*v*) FBS and were incubated for 24 h at 37 °C in serum-free DMEM. The culture medium was then harvested and stored at −20 °C until use. For the assay of gelatin zymography, the culture medium of an appropriate volume (adjusted by vital cell number) was electrophoresed in a 10% sodium dodecyl sulfate (SDS)-PAGE gel containing 0.1% (*w*/*v*) gelatin. The gel (MMP-gel) was washed for 30 min at room temperature in a solution containing 2.5% (*v*/*v*) Triton X-100, changed twice, and subsequently transferred to a reaction buffer for enzymatic reaction containing 1% NaN_3_, 10 mM CaCl_2_, and 40 mM Tris-HCl pH 8.0 at 37 °C with shaking overnight (for 15 h). Finally, the MMP-gel was stained for 30 min with 0.25% (*w*/*v*) Coomassie blue in 10% acetic acid (*v*/*v*) and 50% methanol (*v*/*v*) and de-stained in 10% acetic acid (*v*/*v*) and 50% methanol (*v*/*v*). The relative MMP-2 and MMP-9 activities were quantitated by use of Matrox Inspector 2.1 software (Matrox Imaging, Dorval, QC, Canada).

### 3.8. Western Blotting

Expression levels of endogenous nm23-H1 protein were determined by immunoblotting as described previously [5]. Briefly, the medium was removed and cells were lysed with 20% SDS containing 1 mM phenylmethyl sulfonyl fluoride. The lysate was sonicated on ice for 30 s, followed by centrifugation for 30 min at 4 °C. Protein (40 μg) from the supernatant was resolved by SDS-polyacrylamide gel electrophoresis and transferred onto a nitrocellulose membrane. After blocking with TBS buffer (20 mM Tris-HCl, 150 mM NaCl, pH 7.4) containing 5% nonfat milk, the membrane was incubated with anti-nm23-H1 mAb followed by horseradish peroxidase-conjugated antimouse IgG and then visualized by use of an ECL chemiluminescent detection kit (Amersham Co, Bucks, UK).

### 3.9. Statistical Analysis

Values are expressed as means ± SD and were analyzed by using one-way ANOVA followed by unpaired Student’s *t*-test. Difference between two groups was considered significant at * *p* ≤ 0.05, ** *p* ≤ 0.01, and *** *p* ≤ 0.001. 

## 4. Conclusions

The present results demonstrate that MFB has significant anti-invasion and anti-migration activities against SK-Hep-1 cells, a human hepatoma cell line, and that this effect is mainly associated with the induction of nm23-H1 expression and down-regulation of MMP-2 and MMP-9 activity. These findings indicate that MFB is a potent anti-metastatic agent. Further studies are warranted to verify the in vivo significance of these findings.

## Figures and Tables

**Table 1:** Pigment production by *M. purpureus* CWT715 during fermentation for seven days.

Sample	OD_400nm (yellow)_	OD_470nm (orange)_	OD_500nm (red)_
Day 1	1.0	1.0	1.0
Day 2	1.4	1.4	1.4
Day 3	2.3	2.3	2.5
Day 4	4.4	4.7	5.3
Day 5	4.5	5.2	6.0
Day 6	4.7	5.2	5.8
Day 7	4.0	4.6	5.7

## Supplementary Materials

Supplementary materials can be accessed at: http://www.mdpi.com/1420-3049/21/12/1691/s1.

## Abbreviations

DMEM	Dulbecco‘s Minimal Essential Medium
DSR	Distiller’s sorghum residue
ECM	Extracellular matrix
FBS	Fetal bovine serum
LLC	Lewis lung carcinoma
MMP	Matrix metalloproteinase
MFB	*Monascus purpureus*-fermented broth
MP	*Monascus purpureus* CWT715
SDS	Sodium dodecyl sulfate
