# Supplementary material for: Extract of Monascus purpureus CWT715 Fermented from Sorghum Liquor Biowaste Inhibits Migration and Invasion of SK-Hep-1 Human Hepatocarcinoma Cells
Source: Molecules. 2016 Dec 8;21(12):1691. doi: 10.3390/molecules21121691 (PMC6272986; doi:10.3390/molecules21121691)
Supplement: Supplementary file 1 [file molecules-21-01691-s001.pdf]

Molecules **2016**, *21*, 1691; doi:10.3390/molecules21121691

Supplementary Materials: Extract of *Monascus purpureus* CWT715 Fermented from Sorghum Liquor Biowaste Inhibits Migration and Invasion of SK-Hep-1 Human Hepatocarcinoma Cells

Wen-Teish Chang, Cheng-Hung Chuang, Wan-Ju Lee and Chin-Shiu Huang

(**A**) (**B**)

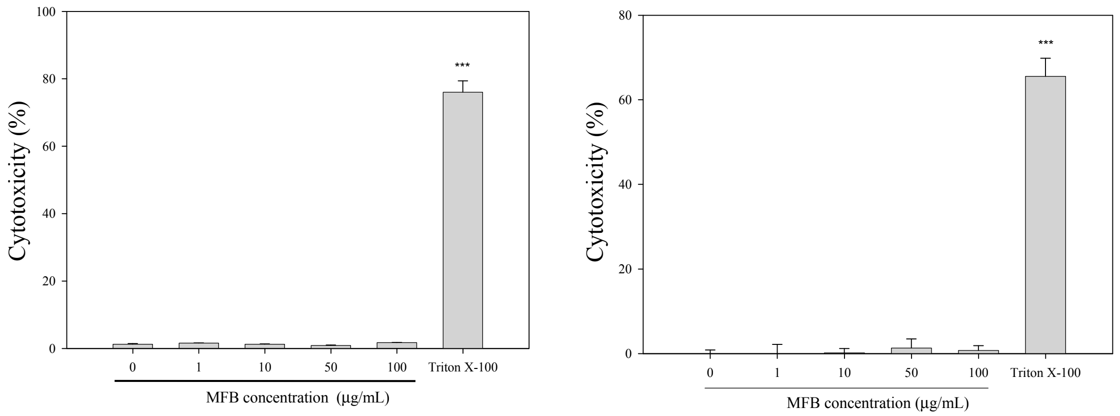

Figure S1. Effects of MFB on the release of LDH in SK-Hep-1 cells. Cells (1 × 10^4) were incubated with different concentration (1–100 μg/mL) of MFB for 24 h. MFB obtained from 6-day fermentation (**A**) and from 7-day fermentation (**B**) were measured the cytotoxicity. Values are means ± SD, $n = 3$; significant difference from control value was indicated *** $p \leq 0.001$.
